# Evaluation of the Antioxidant Properties of Carvacrol as a Prospective Replacement for Crude Essential Oils and Synthetic Antioxidants in Food Storage

**DOI:** 10.3390/molecules28031315

**Published:** 2023-01-30

**Authors:** Israel Ehizuelen Ebhohimen, Ngozi P. Okolie, Moses Okpeku, Mfon Unweator, Victoria T. Adeleke, Lawrence Edemhanria

**Affiliations:** 1 Department of Biochemistry, Ambrose Alli University, Ekpoma 310006, Nigeria; 2 Department of Biochemistry, University of Benin, Benin City 300213, Nigeria; 3 Discipline of Genetics, School of Life Sciences, University of KwaZulu-Natal, Durban 4041, South Africa; 4 Department of Chemical Sciences, Glorious Vision University, Ogwa 310107, Nigeria; 5 Department of Chemical Engineering, Mangosuthu University of Technology, Umlazi 4031, South Africa

**Keywords:** carvacrol, antioxidant, butylated hydroxytoluene, lipoxygenase, molecular docking, molecular dynamics simulation

## Abstract

The phenolic structural analogues of synthetic antioxidants such as butylated hydroxytoluene (BHT) in essential oils have been reported to exhibit antioxidant properties. Additionally, their lipophilicity makes them suitable for use in lipid-rich foods. This study evaluated the antioxidant capacity of carvacrol, a monoterpenoid antioxidant compound in the *Monodora myristica* (Gaertn.) seed essential oil, compared to the seed essential oil and BHT. In vitro studies (ferric reducing antioxidant power (FRAP), metal chelating activity (MCA), and nitric oxide scavenging activity (NOSA)) were conducted to ascertain if the antioxidant capacity of carvacrol was comparable to that of the seed essential oil. The potential binding affinity and molecular interactions between carvacrol and lipoxygenase (LOX) and its homologous model were investigated in silico. The molecular docking was performed using Autodock Vina, and the best poses were subjected to molecular dynamics simulation. The IC_50_ for MCA and NOSA were: carvacrol 50.29 µL/mL, seed essential oil (SEO) 71.06 µL/mL; and carvacrol 127.61 µL/mL, SEO 165.18 µL/mL, respectively. The LOX model was Ramachandran favoured (97.75%) and the overall quality factor in the ERRAT plot was 95.392. The results of the molecular docking and molecular dynamics simulations revealed that lipoxygenase has a higher affinity (−22.79 kcal/mol) for carvacrol compared to BHT. In the LOX–BHT and LOX–carvacrol complexes, the root-mean-square deviation (RMSD), root-mean-square fluctuation (RMSF), and the radius of gyration (RoG) were not significantly different, indicating similar molecular interactions. The results obtained from this study suggest that carvacrol exhibits an antioxidant capacity that may be explored as an alternative for crude essential oils and synthetic compounds during the storage of lipid-rich foods.

## 1. Introduction

Synthetic phenolic antioxidants such as butylated hydroxytoluene (BHT) are commonly used as food additives to prolong shelf life. There is a growing concern about their toxicity, and thus an increased research interest in natural compounds as alternatives [1]. Several studies have revealed the adverse effect of exposure to BHT at doses ranging from 0.5 to 1.0 g/kg. Butylated hydroxytoluene has been reported to promote tumours, although it is anticarcinogenic and has no effect on other carcinogenic agents [2,3,4]. Based on these toxic effects, the FAO prohibited the use of butylated hydroxyanisole (BHA) and BHT in 1980 [5]. The continued use of BHT as a food additive was later recommended since it was only required at low concentrations, and because it did not exhibit the full profile of recognized human carcinogens. Recent studies in animal models now confirm the toxicity of these synthetic phenolic antioxidants. Notably, these compounds can bioaccumulate [5,6,7,8,9]. Researchers at the University of California uncovered 109 new compounds through the suspicious screening of about 3500 industrial chemicals in thirty paired maternal and cord serum samples. The researchers reported that there is a dearth of information about 55 compounds out of the 109 discovered [9].

Several scientific articles on the use of essential oils in food preservation have been published to date [10,11]. The current research interest is on the observed bactericidal, viricidal, fungicidal, antiparasitic, insecticidal, therapeutic, and aesthetic effects of essential oils. The application of essential oils in foods to extend shelf life is mostly based on their antioxidant and antimicrobial properties [8,11]. Although essential oils are fascinating alternatives to chemical preservatives, their usage is constrained by their high volatility, potent odour, and diverse compositions. When the essential oils are applied directly to the food matrix, the change in organoleptic characteristics is more noticeable [10]. Recent innovations to maximize applicability in food systems include encapsulation and inclusion into edible films with controlled release [3,12,13]. The main problem with the use of essential oils as antioxidants is the non-uniformity of the outcomes from their application [14,15]. The lipo-stable constituents of essential oils, which are responsible for the antioxidant property of essential oils, are hypothesized as suitable and safer alternatives for crude essential oils and synthetic compounds [15].

Ebhohimen et al. previously evaluated the biological activities as well as the pharmacokinetic parameters of the constituents of the *Monodora myristica* (Gaertn.) seed essential oil in silico [14]. The outcome of that research indicated that carvacrol, a monoterpenoid, is an antioxidant compound in the seed essential oil. This is consistent with Brewer’s report on the compound’s antioxidant potential. Carvacrol and other phenolic analogues have been reported to exhibit antioxidant potential that can be studied for their application in food preservation [16,17]. The lipo-stability of these compounds can enhance their application in lipid-rich foods such as meat and fish, which are susceptible to post-mortem oxidative reactions due to factors that include the presence of Fe^3+^ in haemoglobin, lipoxygenase activity, and physical tissue damage during slaughter [1,16,18].

This study aimed to determine if carvacrol’s antioxidant capacity is comparable to that of the seed essential oil, and to study the molecular interactions with lipoxygenase compared to the standard synthetic antioxidant, BHT. The application of single bioactive components of crude essential oils will standardize their application as food additives, as they may be safer alternatives to synthetic antioxidants. 

## 2. Results

### 2.1. Molecular Interaction between Carvacrol, BHT, and Lipoxygenase (LOX)

The molecular interactions were studied by molecular docking and molecular dynamics simulation of LOX and its homologous model complexed with carvacrol and BHT. 

#### 2.1.1. Multiple Sequence Alignment (MSA)

The result of MSA analysis of the homologous sequences of LOX downloaded from NCBI revealed several non-conserved regions (Figure S1 from Supplementary Materials).

#### 2.1.2. Predicted Stable Amino Acids in Non-Conserved Regions

The LOX homologous model was built based on the new sequence generated by amino acid substitutions using the Cologne University Protein Stability Analysis Tool (CUPSAT) and 3V92_B as a reference structure. The results are presented in Table 1.

#### 2.1.3. Homology Modelling of LOX

**Figure 1:**
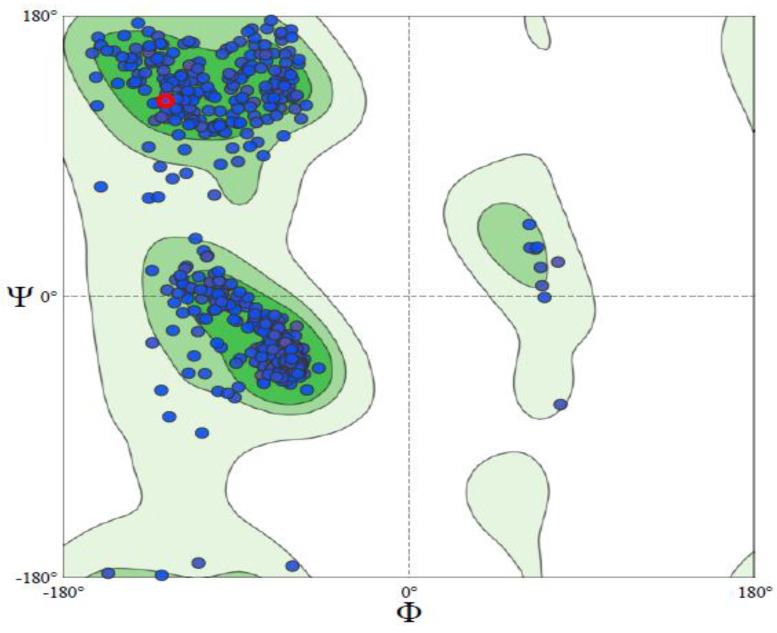
Ramachandran plot for model LOX structure.

**Figure 2:**
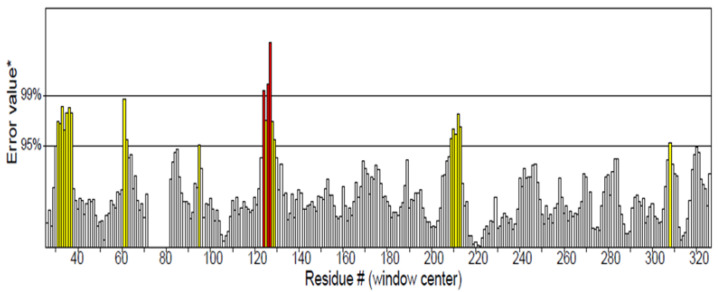
ERRAT plot for model LOX structure [Program: ERRAT2, Overall quality factor *: 95.392].

The homologous model of LOX was built using the SwissModel web server to accommodate possible alterations in molecular interactions that may be induced by amino acid substitutions in other variants of LOX. The structure was authenticated by its Ramachandran score and ERRAT Plot (Figure 1 and Figure 2). The Ramachandran plot enables the visualization of energetically favoured regions for backbone dihedral angles of amino acids in the protein structure, thus being used for structure validation. The MolProbity score provides a single number that represents the quality statistics, including the clashscore, the percentage of Ramachandran score not favoured, and the percentage of bad side-chain rotamers, of a protein structure [19,20]. The MolProbity score for the LOX model was 0.83, and Ramachandran favoured was 97.75%. 

#### 2.1.4. Molecular Docking

**Figure 3:**
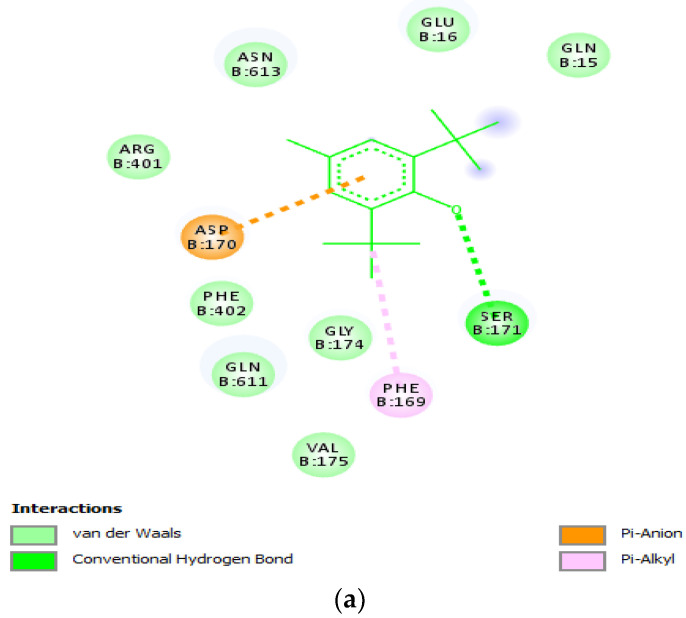

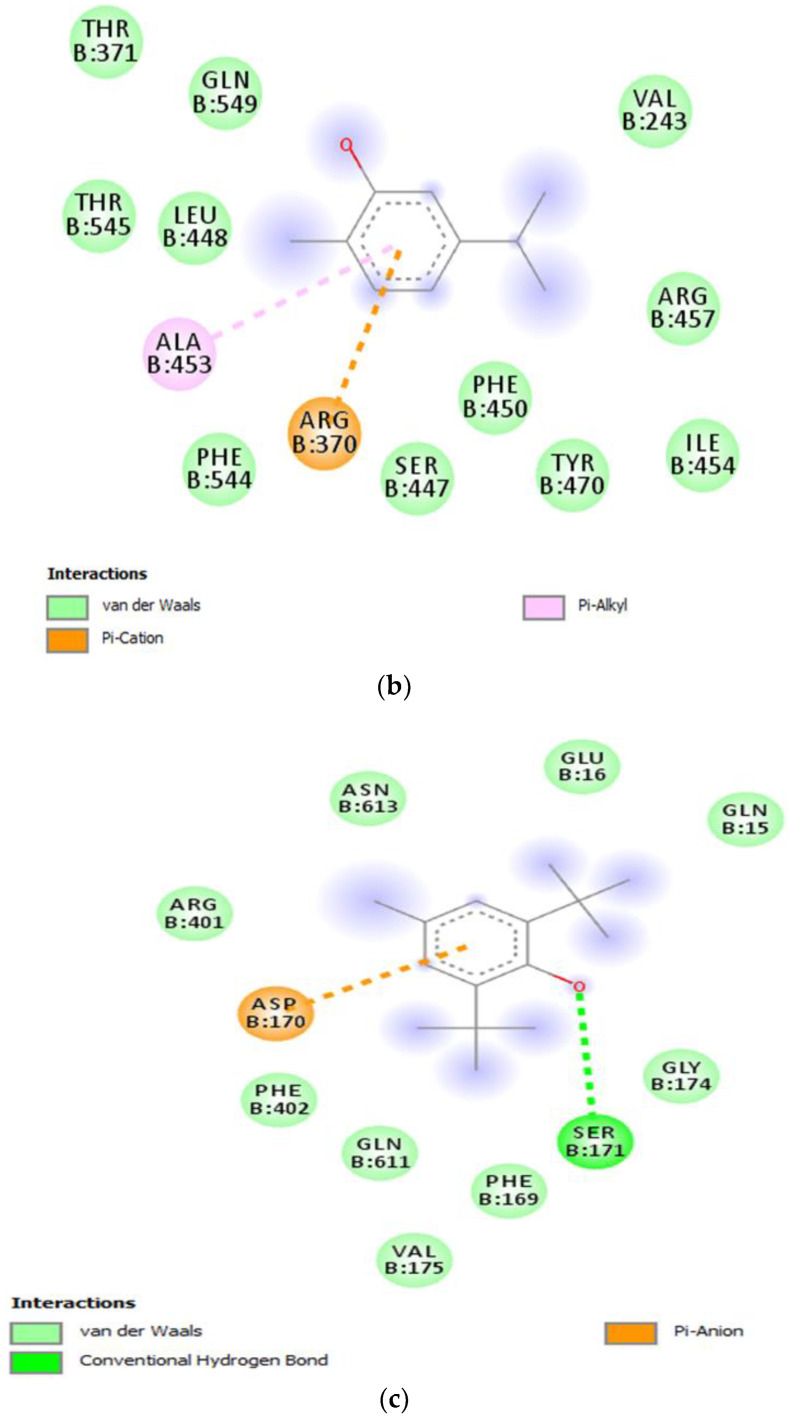

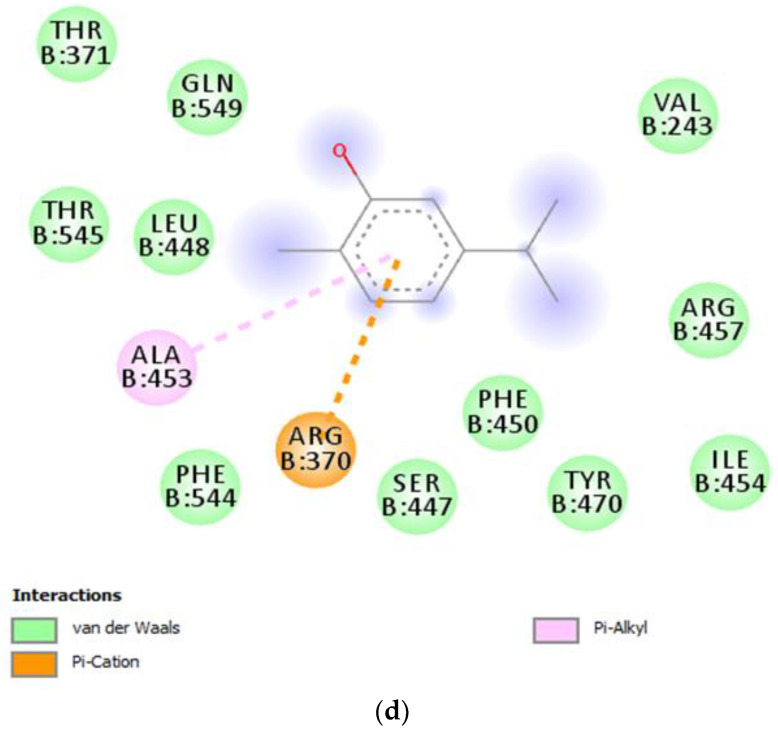
Docked complexes using AutoDock Vina: (**a**) LOX–BHT complex; (**b**) LOX–carvacrol complex; (**c**) LOX model–BHT complex; (**d**) LOX model–carvacrol complex; letter codes are amino acids; B—chain B of LOX [PDB ID: 3V92]; numbers are positions of the amino acids in the peptide sequence.

The results of the molecular docking on AutoDock Vina showed nine different poses with varying RMSD values (Table 2). The best poses for BHT and carvacrol docked on LOX and its homologous model showing the interacting amino residues in the docked complexes are presented in Figure 3a–d.

#### 2.1.5. Molecular Dynamics Simulation

**Figure 4:**
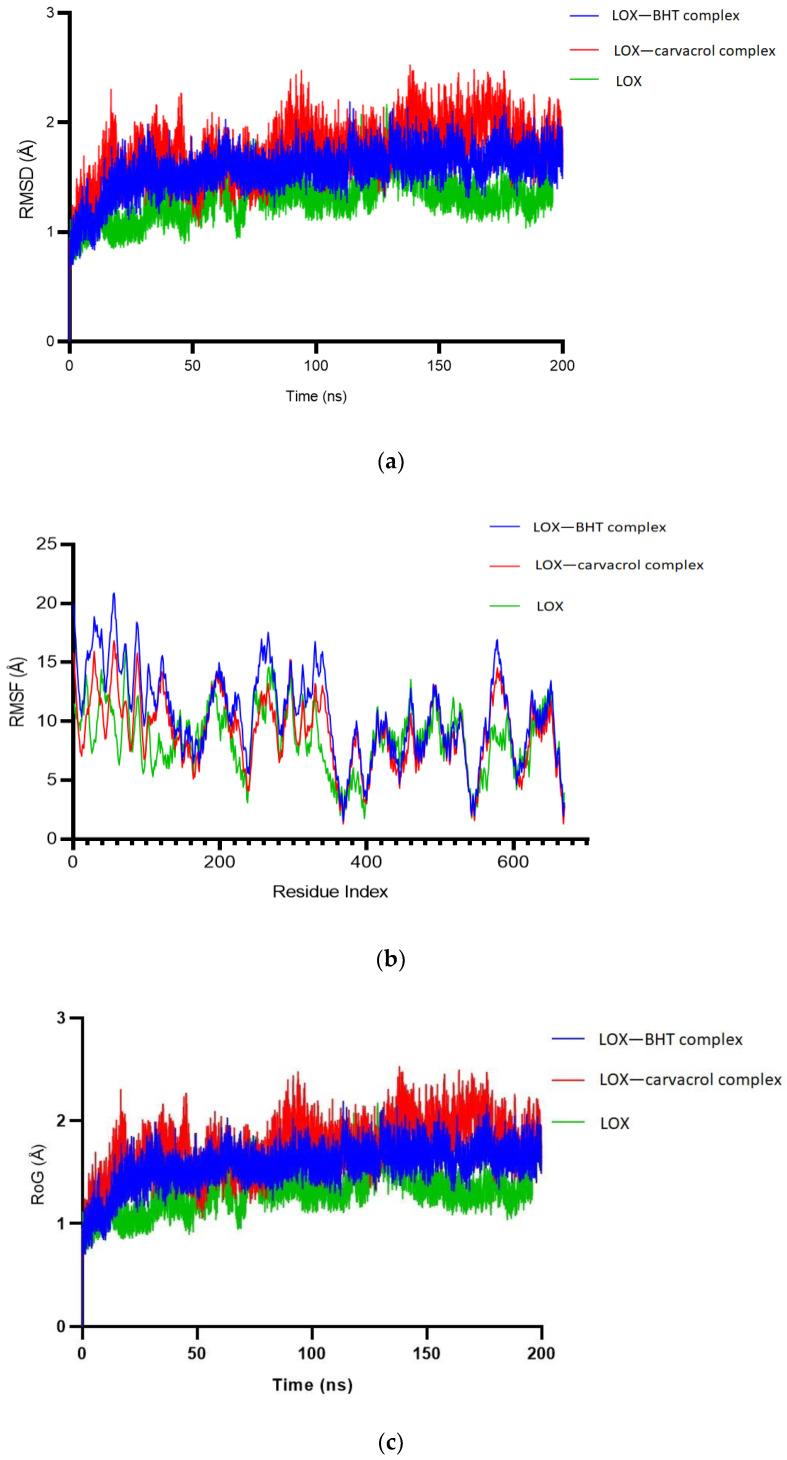
Molecular dynamics simulation of carvacrol and BHT docked on LOX, and LOX in the free state, for 200 nanoseconds; (**a**) root-mean-square deviation (RMSD); (**b**) root-mean-square fluctuation (RMSF); and (**c**) radius of gyration (RoG).

The RMSD ranges for the 200 ns simulation for bound and free LOX indicate that binding to BHT resulted in a slightly more stable complex than binding to carvacrol (Figure 4a). The RMSF values obtained for the LOX in the bound and free states are presented in Figure 4b. Although the atomic fluctuations in both states followed a similar pattern, the range was significantly different between the bound and free states at residues 1–240 with the RMSF values in the free state lower than in the bound state. The RoGs of bound and free LOX were not significantly different, indicating the stability of the bound complexes (Figure 4c). The average values for RMSD, MSF, and RoG in the bound and free states are presented in Table 3. 

### 2.2. In Vitro Antioxidant Capacity of Carvacrol Compared to Seed Essential Oil

#### 2.2.1. Ferrous Metal Chelating Activity of Carvacrol Compared to Seed Essential Oil

**Figure 5:**
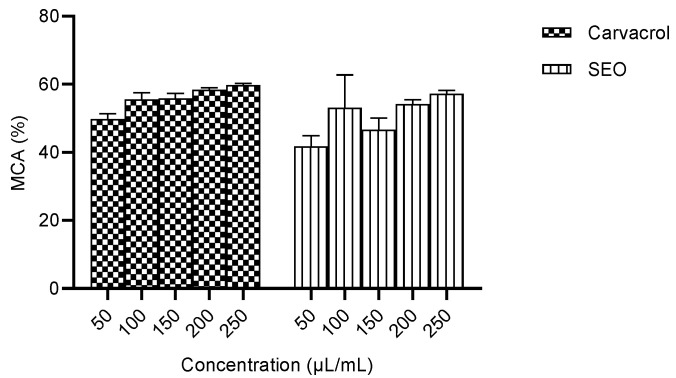
Percentage of ferrous metal chelating activity (IC_50_: carvacrol = 50.29 µL/mL. SEO = 71.06 µL/mL). MCA—metal chelating activity; SEO—seed essential oil.

The highest metal chelating activity was observed at 250 µL/mL. There was no significant difference (*p* > 0.05) between the metal-chelating activities of carvacrol and the seed essential oil (Figure 5).

#### 2.2.2. Nitric Oxide Scavenging Activity of Carvacrol Compared to Seed Essential Oil

**Figure 6:**
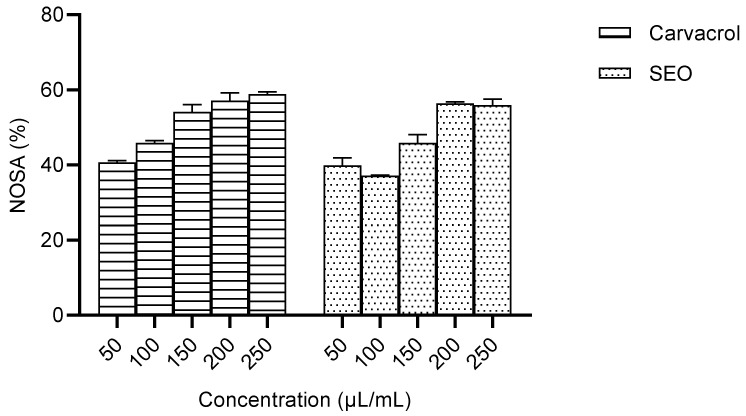
Percentage of nitric oxide scavenging activity [IC_50_ values: carvacrol = 127.61 µL/mL; SEO = 165.18 µL/mL]; SEO—seed essential oil; NOSA—nitric oxide scavenging activity.

The nitric oxide scavenging activities of carvacrol and the seed essential oil were concentration-dependent, but there was no significant difference across the concentrations studied (Figure 6).

#### 2.2.3. Ferric Reducing Power of Carvacrol Compared to Seed Essential Oil

**Figure 7:**
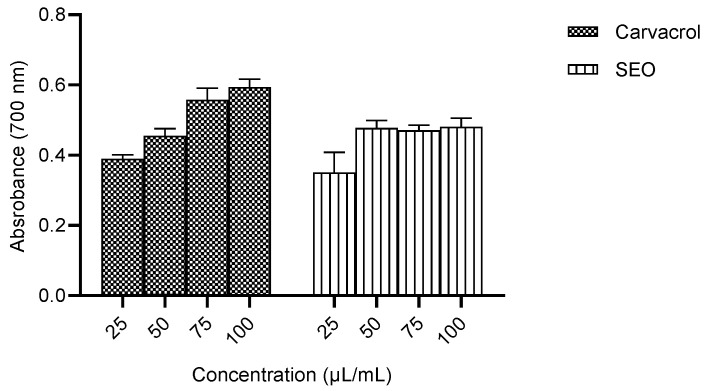
Ferric reducing power. SEO—seed essential oil.

The ferric reducing power of seed essential oil was not concentration-dependent. The reducing power was highest at 100 µL/mL carvacrol, but it was not significantly different from the values obtained for the seed essential oil (Figure 7).

### 2.3. Retention of Carvacrol and BHT after Thermal Treatment

The residual concentration of carvacrol was compared to BHT to ascertain the impact of thermal exposure. The percentage areas for carvacrol and BHT were 37.32 and 53.09%, respectively, with carvacrol having a lower retention time. The GC-MS results are presented in Table 4 and Table 5.

## 3. Discussion

Despite technological advancements in food processing and preservation techniques, lipid peroxidation remains a concern in foods with high lipid content. Food additives as antioxidants have gained widespread use, but there are safety and toxicity concerns. Essential oils have been studied for their use as food additives due to their reported antioxidant and antimicrobial properties [10]. However, the available data indicate quantitative differences in their phytochemical composition, since it is influenced by the prevailing environmental factors [21,22,23]. This variability in the composition and concentration of phytochemicals remains a crucial factor limiting their direct application as food additives [24]. To overcome this limitation, the bioactivities of the active components in crude essential oils are being explored. The antioxidant capacity of carvacrol, a component of the *Monodora myristica* (Gaertn.) seed essential oil, has been previously reported by Ebhohimen et al. [14]. 

The aim of this study was to evaluate the in vitro antioxidant capacity of carvacrol compared to the seed essential oil using some parameters implicated in the onset and propagation of lipid peroxidation in food during storage. The molecular interactions between carvacrol and LOX, an enzyme implicated in the onset of oxidative reactions after harvest, was studied in silico. Furthermore, the impact of thermal treatment on the residual concentration of carvacrol and BHT was investigated. The biological role of LOX in living tissue is the synthesis of 5-hydroperoxy-6,8,11,14-tetraenoic acid, an intermediate in the synthesis of leukotrienes and lipoxins. [25,26]. The enzyme can function post-harvest/slaughtering to initiate lipid peroxidation, thus providing precursors for the chain reaction during storage. The presence of metal ions and other cofactors that stimulate the lipoxygenase reaction further encourages the oxidative process [27,28]. The inhibition of the lipoxygenase action in food is an important strategy to halt enzyme-induced lipid peroxidation, and computational methods are efficient tools to study molecular interactions. 

To improve the outcome of this study, several LOX sequences from the BOS family were obtained from the National Center for Biotechnology Information’s database, and multiple sequence alignment was conducted against human 5-lipoxygenase to identify conserved and non-conserved amino acids [29]. Amino acid substitutions affect the function and stability of enzymes, especially if they occur at the active site. The results of the multiple sequence alignment revealed amino acid substitutions in LOX variants. The favourable and stable amino acids that could substitute non-conserved amino acids were identified (Table 1) using the Cologne University Protein Stability Analysis Tool (CUPSAT) [30,31]. The program uses structural environment-specific atom potentials and torsion angle potentials to predict the difference in free-energy change when a non-conserved amino acid is present and when substituted by the other nineteen amino acids. It requires the crystal structure of the protein in Protein Data Bank format and the location of the residue to be mutated [32]. The more stable amino acids (Table 1) were substituted into the LOX sequence to build a homologous model to gain insight into the possible molecular interactions in LOX complexes with antioxidant compounds when amino acid substitutions occur. Out of the 16 non-conserved amino acids identified in the LOX sequences studied, no amino acids were predicted for LEU127, VAL262, CYS264, SER271, LEU272, and LEU289 (Table 1). Based on the predicted stable amino acids in the non-conserved regions, a homologous model was built using the SwissModel web server. The quality of the predicted structure by homology modelling is an important parameter and it depends on the degree of similarity between the template and model sequences. A low degree of similarity between the sequences yields a low-quality structure [33]. The quality of the 3D structure of the model was ascertained using the model’s stereochemistry, geometry, and other structural properties. The best of these is the Ramachandran plot of the protein’s ψ–ϕ main-chain torsion angles, which identify proteins with numerous outlying residues [34]. Low sequence similarity and high structural divergence indicate the models contain errors. The LOX model in this study was Ramachandran favoured (97.5%), with only one bad bond and 0.15% amino residues as outliers. The MolProbity score was low, indicating a high-quality structure. The quality of the 3D structure of the model was further ascertained using the ERRAT plot to show error values for residues in the amino acid sequence. The *Y*-axis represents the error value, and the *X*-axis represents the residues of the protein model. An error value that exceeds 99% indicates a poorly-modelled region. The overall ERRAT score for the LOX model was 95.39%, confirming the quality of the structure [35].

The molecular docking results from AutoDock Vina (Figure 3a–d) indicated that LOX and its homologous model had a higher affinity for carvacrol (Table 2), which was further confirmed during the molecular dynamics simulation (Table 3). The predicted higher binding affinity may be a function of the lower molecular weight and size of carvacrol compared to BHT. To aid a proper understanding of the molecular interactions, the free protein and the bound complexes were subjected to the same experimental conditions. The positional divergence of one or multiple atoms measured as root-mean-square deviation (RMSD) is one of the most commonly used plot types in the field of biophysical simulations [36]. The observed range of RMSD for the complexes was slightly higher than that of the free LOX, but it was not significantly different. The comparable RMSD values for both carvacrol and BHT indicate that molecular interactions with LOX are stable (Figure 4a). The root-mean-square fluctuation (RMSF) represents the degree of variation of a given atom over time. The RMSF values were plotted per residue for LOX in the bound and free states. Atomic fluctuations varied significantly in both bound and free states between residues 1 and 180. The RMSF for LOX bound to BHT and for carvacrol were very similar between residues 380 and 670 (Figure 4b). The RoG was also studied to determine dynamic adaptability and compactness in an aqueous environment. The RoG for all complexes was similar, indicating their relative stability. The simulation results suggest very stable molecular interactions between LOX–BHT as well as LOX–carvacrol (Figure 4c).

The in vitro studies were conducted to ascertain the antioxidant capacity of carvacrol compared to the crude seed essential oil by measuring the metal chelating activity, ferric ion reducing power and nitric oxide scavenging activity. The results obtained for metal chelating activity were not significantly different, and the IC_50_ for carvacrol was lower than that for the SEO. The metal chelating activity was not significantly different across the concentration range studied for carvacrol, suggesting bioactivity at low concentrations (Figure 5). The capacity to scavenge NO was not significantly different between carvacrol and the SEO, and the IC_50_ for carvacrol was lower than that for the SEO. (Figure 6). The percentage of the ferric reducing power of carvacrol was concentration-dependent, but the activity in the SEO group was not significantly affected by concentration. (Figure 7). The observed lower IC_50_ for carvacrol compared to the SEO indicates a higher antioxidant capacity [37].

The results obtained after the thermal exposure (100 °C) of 2% solutions of carvacrol and BHT revealed a lower retention time and percentage area for carvacrol compared to BHT. The temperature was selected hypothetically to mimic the minimum possible temperature that foods may be exposed to during cooking. The lower molecular weight and size of carvacrol may be responsible for the observations after the thermal treatment (Table 4 and Table 5) of the surface-accessible regions on LOX (Figure 3 and Figure 4). This could be a great advantage when the compound is used for the storage of foods that require thermal processing, as the compound could completely evaporate, thus reducing the possibility of bioaccumulation.

## 4. Materials and Methods

### 4.1. Materials

The gas chromatography–mass spectrometry (GC-MS) reports on the chemical composition of *Monodora myristica* (Gaertn.) seed essential oil by Koudou et al. and Akise et al. were used as sources of chemical compounds that were screened for biological activity [23,38]. Carvacrol, which was identified as an active antioxidant component, was purchased from Sigma Aldrich, Darmstadt, Germany [14]. The chemicals and reagents used for this study were of analytical grade. 

### 4.2. Data Retrieval

The 3D structure of human arachidonate 5-lipoxygenase (LOX) [Protein Data Bank (PDB) ID: 3V92/EC: 1.13.11.34] was downloaded with its FASTA sequence at a resolution of 2.74 Å from https://www.rcsb.org/structure/3V92 (accessed on 20 August 2021). The FASTA sequence was used as a reference sequence for Basic Local Alignment Search Tool-Protein (BLAST-P) (https://blast.ncbi.nlm.nih.gov/Blast.cgi?PROGRAM%20=blastp&PAGE_TYPE=BlastSearch&LINK_LOC=blasthome, accessed on 20 August 2021) to obtain homologous sequences. The search was constrained to the BOS family only, and the seqdump file was downloaded from the National Center for Biotechnology Information (NCBI) Adatabase. 

### 4.3. Multiple Sequence Alignment and Prediction of Stable Amino Acids in Non-Conserved Regions

The multiple sequence alignment was performed using the Clustal Omega web server (https://www.ebi.ac.uk/Tools/msa/clustalo/, accessed on 20 August 2021) to identify conserved and non-conserved amino acids in the peptide sequence. The non-conserved amino acids in the peptide sequence obtained from the MSA were predicted using Cologne University Protein Stability Analysis Tool (CUPSAT) (http://cupsat.tu-bs.de/, accessed on 20 August 2021). A homologous model of the enzyme was built based on the observed stable and favourable amino acids in the non-conserved regions.

### 4.4. Homology Modelling

The stable amino acids were inserted into the LOX peptide sequence, and a homologous model was built using 3V92 as a reference structure on the SwissModel web server https://swissmodel.expasy.org/ (accessed on 28 August 2021).

### 4.5. Docking of Carvacrol and BHT on LOX

The calculation of the binding affinity of carvacrol and BHT to LOX and the homologous model was performed using AutoDock Vina [39]. For the docking, separate receptor and ligand files were prepared using Biovia Discovery Studio 2020. Water molecules and heteroatoms were removed from the crystallographic structure of LOX, and chain B was used for docking.

### 4.6. Molecular Dynamics Simulation 

The proteins and ligands in the docked complexes were prepared for molecular dynamics simulation using UCSF Chimera 1.14. Molecular dynamics simulation was carried out with the AMBER 14 package. The input topologies were generated using the LEAP module. This was performed by introducing ions into the solvation box of water molecules (8 Å). The energy minimisation to obtain the lowest energy for high-energy configurations in the protein was executed. This step was initially performed with 10,000 steps (500 steepest descents with 9500 conjugate gradient) and followed by full minimization of 2000 steps. The system was gradually heated for 2 ns in a canonical ensemble (NVT) with a Langevin thermostat (from 0 to 300 K). The collision frequency applied to the system was 1.0 ps^−1^, with the density of the water system regulated with 4 ns of NPT simulation. The molecular dynamics production was run at 200 ns of NPT (constant number N, pressure P, and temperature T), where equilibration of the system was reached at 300 K for another 2 ns at a pressure of 1 bar. After molecular dynamics simulation, the PTRAJ and CPPTRAJ modules in AMBER 14 were used to analyse root-mean-square deviation (RMSD), root-mean-square fluctuations (RMSF) and region of gyration (RoG) [40].

### 4.7. In Vitro Antioxidant Capacity 

#### 4.7.1. Ferric Reducing Power Assay

The ferric reducing ability was measured as described by Benzie and Strain [41]. Ferric iron (Fe^3+^) is reduced to ferrous iron (Fe^2+^) by accepting electrons. The iron complex formed is a dark-blue coloured solution that absorbs light at 600–700 nm. 

Four dilutions of essential oil and carvacrol were prepared in methanol (25, 50, 75 and 100 μL/mL). Methanol solutions (1 mL) were mixed with phosphate buffer (1 mL, 0.2 M, pH = 6.6) and potassium ferricyanide solution K_3_Fe(CN)_6_ (1 mL, 1%). After incubation for 20 min at 50 °C, trichloroacetic acid (1 mL, 10%) was added and the solution was centrifuged at 3000 rpm for 10 min. The supernatant (1.5 mL) was mixed with distilled water (1.5 mL) and FeCl_3_ solution (150 μL, 0.1%). The absorbance was measured at 700 nm, a higher absorbance indicating a high reducing power.

#### 4.7.2. Ferrous Ion Scavenging (Metal Chelating) Activity 

The ferrous ion scavenging activity was described by Dinis et al. [42]. The presence of chelating agents interrupts the formation of the Fe^2+^ and 1,10-phenanthroline complex, thus producing a decrease in the intensity of the coloured solution.

A reaction mixture containing 0.5 mL of the sample at various concentrations (viz., 30, 60, 90, 120, and 150 µL/mL), 1.6 mL of deionized water, 0.05 mL of FeCl_2_ (2 mM), and 0.1 mL of 1,10-phenanthroline (5 mM) was incubated at 40 °C for 10 min. The absorbance of the solution was measured at 562 nm, and the percentage chelating activity was calculated using the formula:

Metal chelating activity (%) = [1 − (A_1_ − A_2_)/A_0_] × 100 (1)

where A_1_ is the absorbance of the reaction containing all reagents and the sample; A_2_ is the absorbance of the reaction containing the sample and all reagents except for FeCl_2_; and A_0_ is the absorbance of the reaction containing all reagents and FeCl_2_, but not the sample. 

#### 4.7.3. Nitric Oxide Scavenging Assay

The NO scavenging activity was measured using the spectrophotometric principle described by Green et al. [43]. Sodium nitroprusside in an aqueous solution at physiological pH spontaneously generates nitric oxide, which interacts with oxygen to produce nitrite ions that can be estimated spectrophotometrically at 546 nm. 

Sodium nitroprusside solution (1 mL, 5 mM) was mixed with 1 mL of sample at various concentrations (30, 60, 90, 120, and 150 µL/mL). The solution was incubated at 25 °C for 2.5 h. The reaction mixture was mixed with 1 mL of Griess reagent (1% sulphanilamide, 2% phosphoric acid, and 0.1% naphthyl ethylenediamine dihydrochloride), and the absorbance at 546 nm was measured. The percentage of nitric oxide scavenging activity was calculated using the formula:

Nitric oxide scavenging activity (%) = (A_0_ − A/A_0_) × 100 (2)

where A_0_ is the absorbance of the control (all reagents without sample) and Ais the absorbance in the presence of sample.

### 4.8. Retention of BHT and Carvacrol after Thermal Treatment

The residual concentration of BHT and carvacrol was determined by exposing 2% palm olein solutions of carvacrol and BHT to 100 °C in the oven for fifteen minutes separately to ascertain the impact of thermal treatment. The temperature 100 °C was selected as the minimum possible temperature the food material would be exposed to during cooking. Residuals were quantified by percentage area, using GC-MS at the central laboratory, Federal University of Technology, Akure, Ondo State, Nigeria.

The gas chromatographic analysis was performed using Agilent 7890A/5975A GC-MSD system coupled with Agilent 7693A Automatic Liquid Sampler (Agilent Technologies Inc., Santa Clara, CA, USA). A 5% Phenyl Methyl Silox Agilent 19091S-433HP-5MS capillary column (Agilent, Santa Clara, CA, USA) with specification 30 m × 250 µm × 0.25 µm was used. The carrier gas was helium (99.99%) at a flow rate of 1.2 mL/min. The GC oven temperature was kept at 50 °C for 2 min and then programmed to 20 °C/min to 100 °C for 2 min, and then 20 °C/min to 250 °C for 5 min. The injector temperature was 250 °C, while the injection volume was 1 µL with a split ratio of 1:10. The detector temperature was programmed at 300 °C. Mass spectra were recorded at 70 eV. Identification of the fractions was carried out by comparison of their mass spectra with those from NIST 11.

### 4.9. Data Analysis

Data analysis and graphical representations were performed using GraphPad Prism 8.0.2, GraphPad Software, California, USA. The in vitro assays (FRAP, MCA, and NOSA) were conducted in triplicates and reported as means, with *p <* 0.05 taken as statistically significant. The mean and standard error of the mean (SEM) for the data obtained for RMSD, RMSF, and RoG during molecular dynamics simulation were also calculated. 

## 5. Conclusions

The in vitro studies revealed that the monoterpenoid antioxidant compound carvacrol exhibits an appreciable antioxidant capacity compared to the seed essential oil. The molecular interactions between LOX and carvacrol predicted by the molecular docking and molecular dynamics simulation showed that the enzyme has a higher affinity for carvacrol compared to BHT. Furthermore, thermal treatment such as cooking may significantly impact the residual concentration of carvacrol due to its volatility. The bioactivity demonstrated by carvacrol in the in vitro and in silico studies for parameters that are crucial to the onset and propagation of lipid peroxidation in foods suggests that it could be a useful natural substitute for synthetic antioxidants in food storage. The results also suggest that it could be a natural candidate to standardize the application of essential oils as food additives. It is recommended that further research on the effective concentration, the capacity to inhibit lipid concentration in food during storage, and the impact of carvacrol on the organoleptic properties of food be conducted. 

## Figures and Tables

**Table 1:** Stable and favourable amino acid substitutions in non-conserved regions of LOX.

S/N	Residue Present in 3V92	Selected Stable and Favourable Mutation	Amino Acid Letter Code
1	ILE85	CYS	C
2	THR86	TYR	Y
3	ILE95	LYS	K
4	GLY105	ASP	D
5	ASN122	TRP	W
6	ILE124	TRP	W
7	LEU127	^1^ -	^1^ -
8	LYS133	TRP	W
9	TRP144	PRO	P
10	MET231	ILE	I
11	VAL262	^1^ -	^1^ -
12	CYS264	^1^ -	^1^ -
13	ARG268	THR	T
14	SER271	^1^ -	^1^ -
15	LEU272	^1^ -	^1^ -
16	LEU289	^1^ -	^1^ -

^1^ no stable or favourable amino acid is predicted.

**Table 2:** Binding affinity for docked poses using AutoDock Vina.

Mode	Affinity (kcal/mol)			
	BHT–3V92_B	Carvacrol–3V92_B	BHT–model	Carvacrol–model
1	−6.2	−6.9	−5.6	−6.7
2	−6.2	−6.8	−5.6	−6.7
3	−6.2	−6.0	−5.4	−5.8
4	−6.2	−5.9	−5.4	−5.8
5	−6.1	−5.7	−5.4	−5.6
6	−6.1	−5.6	−5.4	−5.5
7	−6.0	−5.6	−5.4	−5.5
8	−6.0	−5.6	−5.3	−5.5
9	−5.9	−5.6	−5.3	−5.4

BHT–3V92_B—docked complex of chain B of 5-lipoxygenase (PDB ID:3V92) and BHT; Carvacrol–3V92_B—docked complex of chain B of 5-lipoxygenase (PDB ID:3V92) and carvacrol; BHT–model—docked complex of LOX model and BHT; Carvacrol–model—docked complex of LOX model and carvacrol.

**Table 3:** Average RMSD, RMSF, and RoG values for LOX in the bound and free state, and binding affinity of docked complexes during molecular dynamics simulation.

	RMSD (Å) (Mean ± SEM)	RMSF (Å) (Mean ± SEM)	RoG (Å) (Mean ± SEM)	Binding Affinity (kcal/mol)
LOX-BHT	1.58 ± 3.12e−4	10.62 ± 0.14	44.30 ± 4.49e−5	−19.71
LOX-carvacrol	1.77 ± 4.57e−4	9.08 ± 0.11	44.30 ± 4.95e−5	−22.79
LOX	1.30 ± 4.33e−4	8.50 ± 0.11	44.29 ± 5.64e−5	

RMSD—root-mean-square deviation; RMSF—root-mean-square fluctuation; RoG—radius of gyration; SEM—standard error of the mean; LOX—lipoxygenase.

**Table 4:** GC-MS analysis of 2% carvacrol solution after thermal treatment for 15 min.

Peak #	Retention Time (Min)	%Composition by Area	Database\NIST11.L: Library/ID	Quality (%)
1	2.199	5.06	1-Propyne	25
2	2.312	4.24	Cyclopropane	49
3	2.475	0.35	Pentane	64
4	2.568	4.4	n-Hexane	43
5	2.625	10.25	Heptane	59
6	3.100	12.55	Cyclohexane	70
7	3.244	2.37	1-Heptene	53
8	3.325	1.02	Cyclopentane	87
9	3.494	2.14	Nonane	64
10	3.582	1.72	3-methyl-Heptane	72
11	3.682	4.19	Cyclohexane	91
12	3.801	2.08	Cyclopentane	94
13	3.901	1.08	Cyclohexane	87
14	3.982	1.52	Cyclohexane	95
15	4.307	0.32	Cyclohexane	87
16	10.425	0.17	2,4-Decadienal	72
17	10.644	0.22	2,4-Decadienal	81
18	10.888	37.32	Carvacrol	60
19	14.084	5.39	Oleic acid	84
20	14.579	2.85	13-octadecadienol	90
21	14.747	0.86	cis-9-Hexadecanoic acid	64

Quality: percentage match of the compounds with library information. GC-MS—gas chromatography–mass spectrometry.

**Table 5:** GC-MS analysis of 2% BHT solution after thermal treatment for 15 min.

Peak #	Retention Time (Min)	%Composition by Area	Database\NIST11.L: Library/ID	Quality (%)
1	2.262	1.27	1-Propyne	17
2	2.343	3.19	Piperazine	45
3	2.675	11.35	Hexane	50
4	3.194	12.63	Cyclohexane	64
5	3.594	1.02	Heptane	59
6	3.663	1.46	4-methyl-Hexane	58
7	3.801	5.95	Cyclohexane	87
8	4.082	1.62	Cyclohexane	94
9	4.439	0.21	3-Ethylcylopentanone	58
10	10.419	−0.28	Oleic acid	50
11	10.775	0.16	2-Hexen-4-yn-1-ol	52
12	11.013	0.16	2,4-Decadienal	74
13	12.446	53.09	Butylated Hydroxytoluene	95
14	14.047	14.53	Oleic acid	93

Quality: percentage match of the compounds with library information. GC-MS—gas chromatography–mass spectrometry.

## Data Availability

The data presented in this study are available in the supplementary material.

## Supplementary Materials

The following supporting information can be downloaded at: https://www.mdpi.com/article/10.3390/molecules28031315/s1, Figure S1: Multiple Sequence Alignment.

## Sample Availability

Samples of the compounds are not available from the authors.
