# Supplementary material for: Evaluation of the Antioxidant Properties of Carvacrol as a Prospective Replacement for Crude Essential Oils and Synthetic Antioxidants in Food Storage
Source: Molecules. 2023 Jan 30;28(3):1315. doi: 10.3390/molecules28031315 (PMC9921622; doi:10.3390/molecules28031315)
Supplement: Supplementary file 1 [file molecules-28-01315-s001.zip › molecules-2170670-supplementary.pdf]

molecules

MDPI

Supplementary Materials

Evaluation of the Antioxidant Properties of Carvacrol as a Prospective Replacement for Crude Essential Oils and Synthetic Antioxidants in Food Storage

Israel Ehizuelen Ebhohimen [1], Ngozi P. Okolie [2], Moses Okpeku [3,*], Mfon Unweator [4], Victoria T. Adeleke [5] and Lawrence Edemhanria [4]

1 Department of Biochemistry, Ambrose Alli University, Ekpoma 310006, Nigeria
2 Department of Biochemistry, University of Benin, Benin City 300213, Nigeria
3 Discipline of Genetics, School of Life Sciences, University of KwaZulu-Natal, Durban 4041, South Africa
4 Department of Chemical Sciences, Glorious Vision University, Ogwa 310107, Nigeria
5 Department of Chemical Engineering, Mangosuthu University of Technology, Umlazi 4031, South Africa
* Correspondence: okpekum@ukzn.ac.za

```
3V92_1|Chains      ------------------------------------------------------------   0
XP_025140741.1     ------------------------------------------------------------   0
XP_006065761.1     ------------------------------------------------------------   0
XP_025140740.1     ------------------------------------------------------------   0
XP_006065760.1     ------------------------------------------------------------   0
XP_006065763.1     ------------------------------------------------------------   0
NP_001179721.1     ------------------------------------------------------------   0
XP_019809802.1     ------------------------------------------------------------   0
CAC33511.1         ------------------------------------------------------------   0
XP_010852818.1     MQGEGTRDRGQEKIFANLEVFTGSQQSVHRGTLPPGILPASAPRHLQPPTPTPTEPQESH   60
XP_005909628.1     -----------------------------------------------PRPPPPTEPQESH   13
MXQ85771.1         ------------------------------------------------------------   0
ELR46226.1         ------------------------------------------------------------   0

3V92_1|Chains      ------------------MGSSHHHHHHSSGLVPRGSHMPSYTVTVATGSQEHAGTDDYI   42
XP_025140741.1     --------------------------------------MPSYTVTVATGSQWFAGTDDYI   22
XP_006065761.1     --------------------------------------MPSYTVTVATGSQWFAGTDDYI   22
XP_025140740.1     --------------------------------------MPSYTVTVATGSQWFAGTDDYI   22
XP_006065760.1     --------------------------------------MPSYTVTVATGSQWFAGTDDYI   22
XP_006065763.1     --------------------------------------MPSYTVTVATGSQWFAGTDDYI   22
NP_001179721.1     --------------------------------------MPSYTVTVATGSQWFAGTDDYI   22
XP_019809802.1     --------------------------------------MPSYTVTVATGSQWFAGTDDYI   22
CAC33511.1         --------------------------------------MPSYTVTVATGSQWFAGTDDYI   22
XP_010852818.1     FPWSRHPGPRIHHTSPHVDFPLWGLRCQQQDACRPHLASVEFS-----------------   103
XP_005909628.1     FPWSRHPGPRIHHTSPHVDFPLRGLRCQQQDACRPHLASVEFSMPHPSGKRALPSHG--V   71
MXQ85771.1         ------------------------------------MP--SYTVTVATGSQWFAGTDDYI   22
ELR46226.1         ------------------------------------------------------------   0

3V92_1|Chains      YLSLVGSAGCSEKHLLDKGS------FERG---------------------------AVD   69
XP_025140741.1     YLSLVGSAGCSEKHLLD---KPFYNDFERGA---------------------------VD   52
XP_006065761.1     YLSLVGSAGCSEKHLLD---KPFYNDFERGA---------------------------VD   52
XP_025140740.1     YLSLVGSAGCSEKHLLD---KPFYNDFERGA---------------------------VD   52
XP_006065760.1     YLSLVGSAGCSEKHLLD---KPFYNDFERGA---------------------------VD   52
XP_006065763.1     YLSLVGSAGCSEKHLLD---KPFYNDFERGA---------------------------VD   52
NP_001179721.1     YLSLVGSAGCSEKHLLD---KPFYNDFERGA---------------------------VD   52
XP_019809802.1     YLSLVGSAGCSEKHLLD---KPFYNDFERGA---------------------------VD   52
CAC33511.1         YLSLVGSAGCSEKHLLD---KPFYNDFERGA---------------------------VD   52
XP_010852818.1     ----------------------------------------------------------VD   105
XP_005909628.1     S---RERAGITDKCTVECDPQPVGPWAGRGLSILQIVDPWGEGGRPQ-----VGKGVPVD   123
MXQ85771.1         YLSLVGSAGCSEKHLLD---KPFYNDFERGA---VRALGWDGLGRPRGRVRARPGPIGVD   76
ELR46226.1         ----------------------------------------------------------VD   2
                                                                             **
```

```
3V92_1|Chains      SYDVTVDEELGEIQLVRIEKRKYGSNDDWYLKYITLKTPHGDYIEFPCYRWITGDVEVVL    129
XP_025140741.1     SYDVTVDEELGDIQLIKIEKRKYWFHDDWYLKYITVKTPCGDYIEFPCYRWISGEGEIVL    112
XP_006065761.1     SYDVTVDEELGDIQLIKIEKRKYWFHDDWYLKYITVKTPCGDYIEFPCYRWISGEGEIVL    112
XP_025140740.1     SYDVTVDEELGDIQLIKIEKRKYWFHDDWYLKYITVKTPCGDYIEFPCYRWISGEGEIVL    112
XP_006065760.1     SYDVTVDEELGDIQLIKIEKRKYWFHDDWYLKYITVKTPCGDYIEFPCYRWISGEGEIVL    112
XP_006065763.1     SYDVTVDEELGDIQLIKIEKRKYWFHDDWYLKYITVKTPCGDYIEFPCYRWISGEGEIVL    112
NP_001179721.1     SYDVTVDEELGDIQLIKIEKRKYWFHDDWYLKYITVKTPCGDYIEFPCYRWISGEGEIVL    112
XP_019809802.1     SYDVTVDEELGDIQLIKIEKRKYWFHDDWYLKYITVKTPCGDYIEFPCYRWISGEGEIVL    112
CAC33511.1         SYDVTVDEELGDIQLIKIEKRKYWFHDDWYLKYITVKTPCGDYIEFPCYRWISGEGEIVL    112
XP_010852818.1     SYDVTVDEELGDIQLIKIEKRKYWFHDDWYLKYITVKTPCGDYIEFPCYRWISGEGEIVL    165
XP_005909628.1     SYDVTVDEELGDIQLIKIEKRKYWFHDDWYLKYITVKTPCGDYIEFPCYRWISGEGEIVL    183
MXQ85771.1         SYDVTVDEELGDIQLIKIEKRKYWFHDDWYLKYITVKTPCGDYIEFPCYRWISGEGEIVL    136
ELR46226.1         SYDVTVDEELGDIQLIKIEKRKYWFHDDWYLKYITVKTPCGDYIEFPCYRWISGEGEIVL    62
                   ***********:***::******  :*********:*** ************:*: *:**

3V92_1|Chains      RDGRAKLARDDQIHILKQHRRKELETRQKQYRWMEWNPGFPLSIDAKCHKDLPRDIQFDS    189
XP_025140741.1     RDGQAKLACDDQIHILKQHRRKELETRQKQYRWMEWNPGFPLSIDAKCHKDLPRDIQFDS    172
XP_006065761.1     RDGQAKLACDDQIHILKQHRRKELETRQKQYRWMEWNPGFPLSIDAKCHKDLPRDIQFDS    172
XP_025140740.1     RDGQAKLACDDQIHILKQHRRKELETRQKQYRWMEWNPGFPLSIDAKCHKDLPRDIQFDS    172
XP_006065760.1     RDGQAKLACDDQIHILKQHRRKELETRQKQYRWMEWNPGFPLSIDAKCHKDLPRDIQFDS    172
XP_006065763.1     RDGQAKLACDDQIHILKQHRRKELETRQKQYRWMEWNPGFPLSIDAKCHKDLPRDIQFDS    172
NP_001179721.1     RDGQAKLACDDQIHVLKQHRRKELETRQKQYRWMEWNPGFPLSIDAKCHKDLPRDIQFDS    172
XP_019809802.1     RDGQAKLACDDQIHVLKQHRRKELETRQKQYRWMEWNPGFPLSIDAKCHKDLPRDIQFDS    172
CAC33511.1         RDGQAKLACDDQIHVLKQHRRKELETRQKQYRWMEWNPGFPLSIDAKCHKDLPRDIQFDS    172
XP_010852818.1     RDGQAKLACDDQIHVLKQHRRKELETRQKQYRWMEWNPGFPLSIDAKCHKDLPRDIQFDS    225
XP_005909628.1     RDGQAKLACDDQIHVLKQHRRKELETRQKQYRWMEWNPGFPLSIDAKCHKDLPRDIQFDS    243
MXQ85771.1         RDGQAKLACDDQIHVLKQHRRKELETRQKQYRWMEWNPGFPLSIDAKCHKDLPRDIQFDS    196
ELR46226.1         RDGQAKLACDDQIHVLKQHRRKELETRQKQYRWMEWNPGFPLSIDAKCHKDLPRDIQFDS    122
                   ***:**** *****:*********************************************

3V92_1|Chains      EKGVDFVLNYSKAMENLFINRFMHMFQSSWNDFADFEKIFVKISNTISERVMNHWQEDLM    249
XP_025140741.1     EKGVDFVLNYSKAMENLFINRFMHMFQSSWSDFADFEKIFVRISNTISERVMNHWQEDLM    232
XP_006065761.1     EKGVDFVLNYSKAMENLFINRFMHMFQSSWSDFADFEKIFVRISNTISERVMNHWQEDLM    232
XP_025140740.1     EKGVDFVLNYSKAMENLFINRFMHMFQSSWSDFADFEKIFVRISNTISERVMNHWQEDLM    232
XP_006065760.1     EKGVDFVLNYSKAMENLFINRFMHMFQSSWSDFADFEKIFVRISNTISERVMNHWQEDLM    232
XP_006065763.1     EKGVDFVLNYSKAMENLFINRFMHMFQSSWSDFADFEKIFVRISNTISERVMNHWQEDLM    232
NP_001179721.1     EKGVDFVLNYSKAMENLFINRFMHMFQSSWSDFADFEKIFVRISNTISERVMNHWQEDRM    232
XP_019809802.1     EKGVDFVLNYSKAMENLFINRFMHMFQSSWSDFADFEKIFVRISNTISERVMNHWQEDRM    232
CAC33511.1         EKGVDFVLNYSKAMENLFINRFMHMFQSSWSDFADFEKIFVRISNTISERVMNHWQEDRM    232
XP_010852818.1     EKGVDFVLNYSKAMENLFINSFMHMFQSSWSDFADFEKIFVRISNTISERVMNHWQEDLM    285
XP_005909628.1     EKGVDFVLNYSKAMENLFINRFMHMFQSSWSDFADFEKIFVRISNTISERVMNHWQEDLM    303
MXQ85771.1         EKGVDFVLNYSKAMENLFINRFMHMFQSSWSDFADFEKIFVRISNTISERVMNHWQEDLM    256
ELR46226.1         EKGVDFVLNYSKAMENLFINRFMHMFQSSWSDFADFEKIFVRISNTISERVMNHWQEDLM    182
                   ******************** *********.**********:**************** *

3V92_1|Chains      FGYQFLNGANPVLIRRCTELPEKLPVTTEMVECSLERQLSLEQEVQQGNIFIVDFELLDG    309
XP_025140741.1     FGYQFLNGCNPVMIQRCLKLPDNLPVTTEMVECSLERQLTLEQEIEQGNIFIVDFKLLDG    292
XP_006065761.1     FGYQFLNGCNPVMIQRCLKLPDNLPVTTEMVECSLERQLTLEQEIEQGNIFIVDFKLLDG    292
XP_025140740.1     FGYQFLNGCNPVMIQRCLKLPDNLPVTTEMVECSLERQLTLEQEIEQGNIFIVDFKLLDG    292
XP_006065760.1     FGYQFLNGCNPVMIQRCLKLPDNLPVTTEMVECSLERQLTLEQEIEQGNIFIVDFKLLDG    292
XP_006065763.1     FGYQFLNGCNPVMIQRCLKLPDNLPVTTEMVECSLERQLTLEQEIEQGNIFIVDFKLLDG    292
NP_001179721.1     FGYQFLNGCNPVMIQRCLKLPDNLPVTTEMVECSLERQLTLEQEIEQGNIFIVDFKLLDG    292
XP_019809802.1     FGYQFLNGCNPVMIQRCLKLPDNLPVTTEMVECSLERQLTLEQEIEQGNIFIVDFKLLDG    292
CAC33511.1         FGYQFLNGCNPVMIQRCLKLPDNLPVTTEMVECSLERQLTLEQEIEQGNIFIVDFKLLDG    292
XP_010852818.1     FGYQFLNGCNPVMIQRCLKLPDNLPVTTEMVECSLERQLTLEQEIEQGNIFIVDFKLLDG    345
XP_005909628.1     FGYQFLNGCNPVMIQRCLKLPDNLPVTTEMVECSLERQLTLEQEIEQGNIFIVDFKLLDG    363
MXQ85771.1         FGYQFLNGCNPVMIQRCLKLPDNLPVTTEMVECSLERQLTLEQEIEQGNIFIVDFKLLDG    316
ELR46226.1         FGYQFLNGCNPVMIQRCLKLPDNLPVTTEMVECSLERQLTLEQEIEQGNIFIVDFKLLDG    242
                   ********.***:*:** :**::****************:****::*********:****
```

```
3V92_1|Chains      IDANKTDPCTLQFLAAPICLLYKNLANKIVPIAIQLNQIPGDENPIFLPSDAKYDWLLAK   369
XP_025140741.1     IDANKTDPCTLQFLAAPICLLYKNLANKIVPIAIQLNQVPGEENPIFLPSDAKYDWLLAK   352
XP_006065761.1     IDANKTDPCTLQFLAAPICLLYKNLANKIVPIAIQLNQVPGEENPIFLPSDAKYDWLLAK   352
XP_025140740.1     IDANKTDPCTLQFLAAPICLLYKNLANKIVPIAIQLNQVPGEENPIFLPSDAKYDWLLAK   352
XP_006065760.1     IDANKTDPCTLQFLAAPICLLYKNLANKIVPIAIQLNQVPGEENPIFLPSDAKYDWLLAK   352
XP_006065763.1     IDANKTDPCTLQFLAAPICLLYKNLANKIVPIAIQLNQVPGEENPIFLPSDAKYDWLLAK   352
NP_001179721.1     IDANKTDPCTLQFLAAPICLLYKNLANKIVPIAIQLNQVPGEENPIFLPSDAKYDWLLAK   352
XP_019809802.1     IDANKTDPCTLQFLAAPICLLYKNLANKIVPIAIQLNQVPGEENPIFLPSDAKYDWLLAK   352
CAC33511.1         IDANKTDPCTLQFLAAPICLLYKNLANKIVPIAIQLNQVPGEENPIFLPSDAKYDWLLAK   352
XP_010852818.1     IDANKTDPCTLQFLAAPICLLYKNLANKIVPIAIQLNQVPGEENPIFLPSDAKYDWLLAK   405
XP_005909628.1     IDANKTDPCTLQFLAAPICLLYKNLANKIVPIAIQLNQVPGEENPIFLPSDAKYDWLLAK   423
MXQ85771.1         IDANKTDPCTLQFLAAPICLLYKNLANKIVPIAIQLNQVPGEENPIFLPSDAKYDWLLAK   376
ELR46226.1         IDANKTDPCTLQFLAAPICLLYKNLANKIVPIAIQLNQVPGEENPIFLPSDAKYDWLLAK   302
                   **************************************:**:******************

3V92_1|Chains      IWVRSSDFHVHQTITHLLRTHLVSEVFGIAMYRQLPAVHPIFKLLVAHVRFTIAINTKAR   429
XP_025140741.1     IWVRSSDFHVHQTITHLLRTHLVSEVFGIAMYRQLPAVHPIFK-----------------   395
XP_006065761.1     IWVRSSDFHVHQTITHLLRTHLVSEVFGIAMYRQLPAVHPIFK-----------------   395
XP_025140740.1     IWVRSSDFHVHQTITHLLRTHLVSEVFGIAMYRQLPAVHPIFKLLVAHVRFTIAINTKAR   412
XP_006065760.1     IWVRSSDFHVHQTITHLLRTHLVSEVFGIAMYRQLPAVHPIFKLLVAHVRFTIAINTKAR   412
XP_006065763.1     IWVRSSDFHVHQTITHLLRTHLVSEVFGIAMYRQLPAVHPIFKLLVAHVRFTIAINTKAR   412
NP_001179721.1     IWVRSSDFHVHQTITHLLRTHLVSEVFGIAMYRQLPAVHPIFKLLVAHVRFTIAINTKAR   412
XP_019809802.1     IWVRSSDFHVHQTITHLLRTHLVSEVFGIAMYRQLPAVHPIFKLLVAHVRFTIAINTKAR   412
CAC33511.1         IWVRSSDFHVHQTITHLLRTHLVSEVFGIAMYRQLPAVHPIFKLLVAHVRFTIAINTKAR   412
XP_010852818.1     IWVRSSDFHVHQTITHLLRTHLVSEVFGIAMYRQLPAVHPIFKLLVAHVRFTIAINTKAR   465
XP_005909628.1     IWVRSSDFHVHQTITHLLRTHLVSEVFGIAMYRQLPAVHPIFKLLVAHVRFTIAINTKAR   483
MXQ85771.1         IWVRSSDFHVHQTITHLLRTHLVSEVFGIAMYRQLPAVHPIFKLLVAHVRFTIAINTKAR   436
ELR46226.1         IWVRSSDFHVHQTITHLLRTHLVSEVFGIAMYRQLPAVHPIFKLLVAHVRFTIAINTKAR   362
                   *******************************************

3V92_1|Chains      EQLICECGLFDKANATGGGGHVQMVQRAMKDLTYASLCFPEAIKARGMESKEDIPYYFYR   489
XP_025140741.1     -----------------------------------------AIKARGMDNAEDIPYYFYR   414
XP_006065761.1     ------------ANATGGGGHVQMVQRAMQDLTYTSLCFPEAIKARGMDNAEDIPYYFYR   443
XP_025140740.1     EQLICEYGLFDK-----------------------------AIKARGMDNAEDIPYYFYR   443
XP_006065760.1     EQLICEYGLFDKANATGGGGHVQMVQRAMQDLTYTSLCFPEAIKARGMDNAEDIPYYFYR   472
XP_006065763.1     EQLICEYGLFDKANATGGGGHVQMVQRAMQDLTYTSLCFPEAIKARGMDNAEDIPYYFYR   472
NP_001179721.1     EQLICEYGLFDKANATGGGGHVQMVQRAMQDLTYTSLCFPEAIKARGMDNAEDIPYYFYR   472
XP_019809802.1     EQLICEYGLFDKANATGGGGHVQMVQRAMQDLTYTSLCFPEAIKARGMDNAEDIPYYFYR   472
CAC33511.1         EQLICEYGLFDKANATGG------------------------------------------   430
XP_010852818.1     EQLICEYGLFDKANATGGGGHVQMVQRAMQDLTYTSLCFPESIKARGMDNAEDIPYYFYR   525
XP_005909628.1     EQLICEYGLFDKANATGGGGHVQMVQRAMQDLTYTSLCFPEAIKARGMDNAEDIPYYFYR   543
MXQ85771.1         EQLICEYGLFDKANATGGGGHVQMVQRAMQDLTYTSLCFPEAIKARGMDNAEDIPYYFYR   496
ELR46226.1         EQLICEYGLFDKANATGGGGHVQMVQRAMQDLTYTSLCFPEAIKARGMDNAEDIPYYFYR   422

3V92_1|Chains      DDGLLVWEAIRTFTAEVVDIYYEGDQVVEEDPELQDFVNDVYVYGMRGRKSSGFPKSVKS   549
XP_025140741.1     DDGLLVWEAIKTFVAEVVDIYYESDQVVEEDQELQDFVKDVYMYGMRGKKASGFPKSIKT   474
XP_006065761.1     DDGLLVWEAIKTFVAEVVDIYYESDQVVEEDQELQDFVKDVYMYGMRGKKASGFPKSIKT   503
XP_025140740.1     DDGLLVWEAIKTFVAEVVDIYYESDQVVEEDQELQDFVKDVYMYGMRGKKASGFPKSIKT   503
XP_006065760.1     DDGLLVWEAIKTFVAEVVDIYYESDQVVEEDQELQDFVKDVYMYGMRGKKASGFPKSIKT   532
XP_006065763.1     DDGLLVWEAIKTFVAEVVDIYYESDQVVEEDQELQDFVKDVYMYGMRGKKASGFPKSIKT   532
NP_001179721.1     DDGLLVWEAIKTFVAEVVGIYYEGDQVVEEDQELQDFVKDVYMYGMRGRKASGFPKSIKA   532
XP_019809802.1     DDGLLVWEAIKTFVAEVVGIYYEGDQVVEEDQELQDFVKDVYMYGMRGRKASGFPKSIKT   532
CAC33511.1         ------------------------------------------------------------   430
XP_010852818.1     DDGLLVWEAIKTFVAEVVDIYYESDQVVEEDQELQDFVKDVYMYGMRGKKASGFPKSIKT   585
XP_005909628.1     DDGLLVWEAIKTFVAEVVDIYYEGDQVVEEDQELQDFVKDVYMYGMRGRKASGFPKSIKT   603
MXQ85771.1         DDGLLVWEAIKTFVAEVVDIYYEGDQVVEEDQELQDFVKDVYMYGMRGRKASGFPKSIKT   556
ELR46226.1         DDGLLVWEAIKTFVAEVVDIYYEGDQVVEEDQELQDFVKDVYMYGMRGRKASGFPKSIKT   482
```

```
3V92_1|Chains     REQLSEYLTVVIFTASAQHAAVNFGQYDWASWIPNAPPTMRAPPPTAKGVVTIEQIVDTL     609
XP_025140741.1    KEKLCEYLTVVIFTASAQHAAVNFGQYDWCSWIPNAPPTMRAPPPTAKGVVTIEQIVETL     534
XP_006065761.1    KEKLCEYLTVVIFTASAQHAAVNFGQYDWCSWIPNAPPTMRAPPPTAKGVVTIEQIVETL     563
XP_025140740.1    KEKLCEYLTVVIFTASAQHAAVNFGQYDWCSWIPNAPPTMRAPPPTAKGVVTIEQIVETL     563
XP_006065760.1    KEKLCEYLTVVIFTASAQHAAVNFGQYDWCSWIPNAPPTMRAPPPTAKGVVTIEQIVETL     592
XP_006065763.1    KEKLCEYLTVVIFTASAQHAAVNFGQ----------------------------------     558
NP_001179721.1    KEKLCEYLTVVIFTASAQHAAVNFGQYDWCSWIPNAPPTMRAPPPTAKGVVTIEQIVETL     592
XP_019809802.1    KEKLCEYLTVVIFTASAQHAAVNFGQYDWCSWIPNAPPTMRAPPPTAKGVVTIEQIVETL     592
CAC33511.1        ------------------------------------------------------------     430
XP_010852818.1    KEKLCEYLTVVIFTASAQHAAVNFGQYDWCSWIPNAPPTMRAPPPTAKGVVTIEQIVETL     645
XP_005909628.1    KEKLCEYLTVVIFTASAQHAAVNFGQYDWCSWIPNAPPTMRAPPPTAKGVVTIEQIVETL     663
MXQ85771.1        KEKLCEYLTVVIFTASAQHAAVNFGQYDWCSWIPNAPPTMRAPPPTAKGVVTIEQIVETL     616
ELR46226.1        KEKLCEYLTVVIFTASAQHAAVNFGQYDWCSWIPNAPPTMRAPPPTAKGVVTIEQIVETL     542

3V92_1|Chains     PDRGRSCWHLGAVWALSQFQENELFLGMYPEEHFIEKPVKEAMARFRKNLEAIVSVIAER     669
XP_025140741.1    PDRGRSCWHLGAVWALSQFQDNELFLGMYPEEHFIEKPVKEAMARFRKNLDSIVSVIAER     594
XP_006065761.1    PDRGRSCWHLGAVWALSQFQDNELFLGMYPEEHFIEKPVKEAMARFRKNLDSIVSVIAER     623
XP_025140740.1    PDRGRSCWHLGAVWALSQFQDNELFLGMYPEEHFIEKPVKEAMARFRKNLDSIVSVIAER     623
XP_006065760.1    PDRGRSCWHLGAVWALSQFQDNELFLGMYPEEHFIEKPVKEAMARFRKNLDSIVSVIAER     652
XP_006065763.1    -----------------------LFLGMYPEEHFIEKPVKEAMARFRKNLDSIVSVIAER     595
NP_001179721.1    PDRGRSCWHLGAVWALSQFQDNELFLGMYPEEHFVEKPVKEAMARFRKNLDSIVSVIAER     652
XP_019809802.1    PDRGRSCWHLGAVWALSQFQDNELFLGMYPEEHFVEKPVKEAMARFRKNLDSIVSVIAER     652
CAC33511.1        ------------------------------------------------------------     430
XP_010852818.1    PDRGRSCWHLGAVWALSQFQDNELFLGMYPEEHFIEKPVKEAMARFRKNLDSIVSVIAER     705
XP_005909628.1    PDRGRSCWHLGAVWALSQFQDNELFLGMYPEEHFVEKPVKEAMARFRKNLDSIVSVIAER     723
MXQ85771.1        PDRGRSCWHLGAVWALSQFQDNELFLGMYPEEHFVEKPVKEAMARFRKNLDSIVSVIAER     676
ELR46226.1        PDRGRSCWHLGAVWALSQFQDNELFLGMYPEEHFVEKPVKEAMARFRKNLDSIVSVIAER     602

3V92_1|Chains     NENLQLPYYYLAPDRIPNSVAI     691
XP_025140741.1    NKNKKLPYYYLSPDRIPNSVAI     616
XP_006065761.1    NKNKKLPYYYLSPDRIPNSVAI     645
XP_025140740.1    NKNKKLPYYYLSPDRIPNSVAI     645
XP_006065760.1    NKNKKLPYYYLSPDRIPNSVAI     674
XP_006065763.1    NKNKKLPYYYLSPDRIPNSVAI     617
NP_001179721.1    NKNKKLPYYYLSPDRIPNSVAI     674
XP_019809802.1    NKNKKLPYYYLSPDRIPNSVAI     674
CAC33511.1        ----------------------     430
XP_010852818.1    NKNKKLPYYYLSPDRIPNSVAI     727
XP_005909628.1    NKNKKLPYYYLSPDRIPNSVAI     745
MXQ85771.1        NKNKKLPYYYLSPDRIPNSVAI     698
ELR46226.1        NKNKKLPYYYLSPDRIPNSVAI     624
```

Figure S1. Multiple sequence Alignment.
